# Association of circadian rhythm genes *ARNTL/BMAL1* and *CLOCK* with multiple sclerosis

**DOI:** 10.1371/journal.pone.0190601

**Published:** 2018-01-11

**Authors:** Polona Lavtar, Gorazd Rudolf, Aleš Maver, Alenka Hodžić, Nada Starčević Čizmarević, Maja Živković, Saša Šega Jazbec, Zalika Klemenc Ketiš, Miljenko Kapović, Evica Dinčić, Ranko Raičević, Juraj Sepčić, Luca Lovrečić, Aleksandra Stanković, Smiljana Ristić, Borut Peterlin

**Affiliations:** 1 Clinical Institute of Medical Genetics, University Medical Centre Ljubljana, Ljubljana, Slovenia; 2 Departments of Biology and Medical Genetics, School of Medicine, University of Rijeka, Rijeka, Croatia; 3 Laboratories of Radiobiology and Molecular Genetics, Institute of Nuclear Sciences “Vinča”, Belgrade, Serbia; 4 Department of Neurology, University Medical Centre Ljubljana, Ljubljana, Slovenia; 5 Departments of Family Medicine, Medical School, University of Ljubljana, Ljubljana, Slovenia; 6 Departments of Family Medicine, Medical School, University of Maribor, Maribor, Slovenia; 7 Departments of Neurology, Military Medical Academy, Belgrade, Serbia; 8 Postgraduate Studies, School of Medicine, University of Rijeka, Rijeka, Croatia; University of Texas Southwestern Medical Center, UNITED STATES

## Abstract

Prevalence of multiple sclerosis varies with geographic latitude. We hypothesized that this fact might be partially associated with the influence of latitude on circadian rhythm and consequently that genetic variability of key circadian rhythm regulators, *ARNTL* and *CLOCK* genes, might contribute to the risk for multiple sclerosis. Our aim was to analyse selected polymorphisms of *ARNTL* and *CLOCK*, and their association with multiple sclerosis. A total of 900 Caucasian patients and 1024 healthy controls were compared for genetic signature at 8 SNPs, 4 for each of both genes. We found a statistically significant difference in genotype (*ARNTL* rs3789327, P = 7.5·10^−5^; *CLOCK* rs6811520 P = 0.02) distributions in patients and controls. The *ARNTL* rs3789327 CC genotype was associated with higher risk for multiple sclerosis at an OR of 1.67 (95% CI 1.35–2.07, P = 0.0001) and the *CLOCK* rs6811520 genotype CC at an OR of 1.40 (95% CI 1.13–1.73, P = 0.002). The results of this study suggest that genetic variability in the *ARNTL* and *CLOCK* genes might be associated with risk for multiple sclerosis.

## Introduction

Multiple Sclerosis (MS) is the most common disabling neurological disease of young adults, starting most often between 20 to 40 years of age. One of the interesting epidemiological characteristics of multiple sclerosis, chronic progressive inflammatory demyelinating disease of the central nervous system, is a gradient of increasing prevalence with geographic latitude, from the Equator to the North and South [1, 2]. Climate, sunlight and day/night dynamics have been investigated as possible causes of the disease [3]. Widely accepted risk factor that contributes to this geographical trend is low vitamin D level. Exposure to sunlight acts protectively against MS by increasing vitamin D levels, however, sun exposure and vitamin D status might independently influence risk for MS [4].

Daily fluctuations of temperature and light intensity and its spectral composition, as well as changes in day length and temperature during different seasons are main factors that maintain the 24-hour period of human circadian rhythms [5]. Circadian rhythms serve to align physiological functions with the environment and are controlled by evolutionarily conserved, self-sustained, yet tuneable, internal clocks. Their main responsibility is to translate the information about time to the organism in such a way, that it can effectively adjust physiological and behavioural responses during the daily cycle. The core regulators are two interlocked transcriptional and post-translational feedback loops, one of them being positive feedback loop through *ARNTL(BMAL1)*/*CLOCK* heterodimers [6, 7]. The circadian clock influences hormonal axes regulation, behaviour, cognitive function, metabolism, cell proliferation, apoptosis, and responses to genotoxic stress and is therefore crucial for optimal health.

Desynchronization of circadian rhythms has been linked to various disorders—neurodegenerative disorders, metabolic disorders, neuropsychiatric diseases, cardiovascular dysfunction, cancer and dysregulation of the immune system [8–12]. Furthermore, working in shifts, which disrupts circadian rhythms and leads to dysregulation of the immune system has been suggested to be associated with higher risk for MS [13, 14].

We therefore hypothesized that genetic variability of key circadian rhythm regulators, *ARNTL* and *CLOCK* genes, might contribute to the risk for MS.

## Methods

### Ethics statement

The study was approved by Slovenian National medical ethics committee (reference number: 98/12/10). The study was conducted according to the Declaration of Helsinki. All patients and controls gave written informed consent to participate in the study.

### Patients

A retrospective cross-sectional case-control genetic association study was performed. A total of 900 patients and 1024 healthy controls were recruited by collaborating genetic centres. Study included patients with definitive MS disease who fulfilled McDonald’s criteria for MS [15]. There were 620 female and 280 male patients. Patients’ details are presented in Table 1. All patients filled up the structured questionnaire about family history and risk factors associated with MS. The control group consisted of ethnically, age- and sex- matched healthy individuals, 476 male and 550 female subjects. All patients and control subjects were Caucasian of the Slavic (Slovene, Croatian and Serbian) origin.

**Table 1:** Clinical characteristics of MS patients (N = 900).

All (N = 900)		Women (N = 620)	Men (N = 280)
Age ^a^	40.76 ± 11.87	41.11 ± 12.40	40.02 ± 10.69
Age at onset ^a^	30.45 ± 9.13	30.53 ± 9.29	30.25 ± 8.80
Course N(%):			
Primary progressive (PP)	48 (5.33%)	28 (4.52%)	20 (7.14%)
Relapsing-remitting (RR)	596 (66.22%)	413 (66.61%)	183 (65.36%)
Secondary progressive (SP)	229 (25.44%)	159 (25.65%)	70 (25.00%)
Benign MS	14 (1.56%)	9 (1.45%)	5 (1.79%)
CIS ^b^	13 (1.44%)	11 (1.77%)	2 (0.71%)
Duration (years) ^a^	10.23 ± 8.64	10.28 ± 8.39	10.13 ± 9.19
EDSS ^a,c^	3.66 ± 2.42	3.70 ± 2.48	3.57 ± 2.29

^a^ mean ± SD

^b^ Clinicaly Isolated Syndrome (CIS)

^a,c^ Disability was assessed by using Kurtzke’s Expanded Disability Status Scale (EDSS).

### Genotyping

Eight tagging single nucleotide polymorphisms (SNPs) were chosen from both genes. SNP’s selection was based on the known genetic linkage in both genes, according to HapMap Phase 3 (http://www.hapmap.org) as previously described [16]. Of these, four intronic SNPs were selected in the *CLOCK* gene (rs11932595, rs6811520, rs6850524, and rs13124436), and four intronic SNPs in the *ARNTL* gene (rs3789327, rs1481892, rs4757144, and rs12363415).

Genomic DNA was isolated from the peripheral blood samples using standard procedures. SNPs genotyping was carried out by real time PCR method performed on 7000 Sequence Detection System (Applied Biosystems, Foster City, CA, USA) using KASPar SNP genotyping chemistry as recommended by manufacturer. The protocol for PCR amplification was as follows: initial denaturation step at 94°C for 15 minutes, followed by 20 cycles of denaturation at 94°C for 10 sec, annealing at 57°C or 61°C for 5 sec, extension at 72°C for 10 sec, and final extension at 72°C for 40 sec.The allelic discrimination analysis was performed using SDS Software Version 1.2 (Applied Biosystems, Foster City, CA, USA).

Genotype assignment was performed and interpreted independently by three investigators.

The STREGA guidelines were followed throughout this study [17].

### Statistical analysis

The significance of the difference of observed alleles and genotypes between MS patients and control subjects, including odds ratios (OR) and their respective 95% confidence intervals (CI), were determined using the Chi-Square test (χ2). Also, deviations from genotype distributions predicted by the Hardy-Wienberg equilibrium were tested using the χ2 test. Calculated associations were regarded as significant when they reached the p≤0.05. Appropriate corrections of significance values for multiple testing were applied using the Benjamini-Hochberg correction method (false-discovery rate–FDR values) as multiple SNPs were analysed.

Haplotype frequencies were estimated using haplo.stats package 16 [18]. The *haplo*.*score* function was used to directly ascertain differences in haplotype distributions across the groups of MS patients and healthy controls. A global test of association and per-haplotype association test were performed for both investigated genes. To reduce the effects of multiple testing and minimize the likelihood of spurious associations among rare haplotypes, we excluded all haplotypes with a frequency below 5% from downstream tests. All employed analyses were performed in R statistical environment (R 2.15.0).

## Results

The MS patient group consisted of 620 females and 280 males, 47.5±26.5years of age at blood sampling. The female to male ratio was 2.21.The control group consisted of 550 female and 476 male subjects of the same ethnic background (mean age 46.5±24.5 years).

The observed distribution of genotypes showed no significant difference when compared with those predicted from the Hardy-Weinberg equilibrium for either patients or controls (P>0.05), with the exception of rs12363415, which was excluded from further analyses. Genotype and allelic distribution of the *ARNTL* and *CLOCK* polymorphisms in MS patients and healthy controls are shown in Table 1.We found a statistically significant difference in the genotype distribution of rs3789327 in *ARNTL* gene (P = 7.5·10^−5^) and rs6811520 in *CLOCK* gene (P = 0.02). As shown in Table 2, the CC genotype of rs3789327 significantly increased risk for MS at an OR of 1.68 (95% CI 1.35–2.07, P = 0.0001). Also, the CC genotype of rs6811520 significantly increased risk for MS at an OR of 1.40 (95% CI 1.13–1.73, P = 0.002).

**Table 2:** Genotypes of circadian rhythm genes for SCS MS consortium.

Genotypes		Total control (%)	Total MS (%)	χ2		P corr.	OR [95% confidence interval]
rs3789327	CC	198 (19.68)	257 (29.11)	22.89		**7.49∙10–5**	CC/CT+TT **1.68 [1.35–2.07], P = 0,0001**
	CT	489 (48.61)	375 (42.47)				
	TT	319 (31.71)	251 (28.43)				
rs1481892	CC	93 (9.57)	73 (9.97)	0.16		1.00	GG/CC+GC 1.02 [0.84–1.23]
	GC	424(43.62)	313 (42.76)				
	CC	455 (46.81)	346 (47.27)				
rs4757144	AA	280 (31.67)	190 (27.54)	3.28		1.00	GG/AA+AG 1.04 [0.81–1.32]
	GA	422 (47.74)	354 (51.30)				
	GG	182 (20.59)	146 (21.16)				
rs6811520	CC	598 (64.09)	512 (72.01)	11.75		**0.02**	CC/CT+TT **1.40 [1.13–1.73], P = 0,002**
	CT	274 (29.37)	166 (23.35)				
	TT	61 (6.54)	33 (4.64)				
rs6850524	CC	151 (15.07)	138 (15.94)	2.38		1.00	GG/CC+GC 1.12 [0.93–1.36]
	GC	502 (50.10)	403 (46.54)				
	GG	349 (34.83)	325 (37.53)				
rs11932595	AA	306 (33.89)	250 (30.38)	4.96		0.59	GG/AA+AG 1.3 [1.02–1.67]
	GA	450 (49.83)	397 (48.95)				
	GG	147 (16.28)	164 (20.22)				
rs13124436	AA	88 (11.47)	83 (10.81)	0.36		1.00	GG/AA+AG 1.06 [0.86–1.29]
	GA	336 (43.81)	331 (43.10)				
	GG	343 (44.72)	354 (46.09)				

We performed analysis for genotype distribution of the selected SNPs in MS patients and controls stratified according to gender. Statistical significance was limited to the female population of MS patients (rs3789327 p-value = 0.007, x^2^ = 15.03; rs6811520 p-value = 0.007, x^2^ = 15.05) while in the male population we have not observed any statistically significant differences in distribution of genotypes/alleles in the ARNTL and CLOCK genes (rs3789327 p-value = 0.06, x^2^ = 9.6; rs6811520 p-value = 3.1, x^2^ = 1.63).

We performed analysis for genotype distribution of the selected SNPs in MS patients and controls according to the age of disease onset, EDSS score, and MS type (PP/RR/SP). We have not observed any statistically significant distributions of polymorphisms in the ARNTL and CLOCK genes according to the age of disease onset and EDSS score.

However, the stratified analysis by clinically defined subtypes of MS has shown a statistically significant difference in the distribution of rs3789327 polymorphism genotypes of the ARNTL gene limited to RR form of MS (P-value 2.5∙10–5, x^2^ = 25.1).

In addition, inferred haplotypes were analysed in both genes. A statistically significant difference in haplotype distribution between the groups of MS patients and healthy controls was found at both gene loci: at *ARNTL* gene locus for haplotype CGG (P = 4.00·10^−3^) and TGA (P = 0.03) and at *CLOCK* gene locus for haplotype TCAG (P = 1.00·10^−3^) (Tables 3 and 4).

**Table 3:** Haplotypes in ARNTL gene.

ARNTL GENE						
rs3789327	rs1481892	rs4757144	Hap freq. (%)	P corr.	% control	% MS
C	C	G	14.95	1.00	14.70	15.20
C	G	A	22.10	0.18	21.02	23.17
C	G	G	8.44	4.00∙10–3	6.93	10.34
T	C	G	11.94	1.31	12.15	11.66
T	G	A	27.61	0.03	29.31	25.58
T	G	G	10.51	1.00	11.40	9.63

**Table 4:** Haplotypes in CLOCK gene.

CLOCK GENE							
rs6811520	rs6050524	rs11932595	rs13124436	Hap freq. (%)	P corr.	% control	% MS
C	C	A	G	15.56	0.23	14.17	17.46
C	C	G	G	5.21	0.53	4.77	5.62
C	G	A	A	19.54	1.00	20.66	18.44
C	G	G	A	12.43	1.00	11.49	13.29
C	G	G	G	24.49	1.00	24.49	24.59
T	C	A	G	18.14	1.00∙10–3	20.68	14.99

## Discussion

We hypothesized that genetic variation in the key genes regulating circadian rhythm might contribute to the MS risk. Namely, genetic epidemiology data demonstrated significantly different allele, genotype and haplotype distribution of *CLOCK* gene among worldwide populations, potentially interesting for health association studies [19]. To our knowledge, this is the first report on association between genetic variability of key circadian rhythm regulators, *ARNTL* and *CLOCK* genes, and multiple sclerosis risk.

There are several lines of evidence supporting the involvement of circadian rhythm in the pathogenesis of MS. Studies have shown that shift work at young age increases the risk for MS [13]. Besides sleep restriction/deprivation, shift work also disrupts circadian cycles, and these both lead to melatonin secretion disturbances and augmented pro-inflammatory activity and could be a factor in the inflammatory reactions in the pathophysiologic process of MS. Melatoninis directly involved in circadian and seasonal rhythms and it exerts anti-inflammatory effects through restraining the production of pro-inflammatory cytokines [20, 21]. The correlation between melatonin secretion disturbances and MS have already been shown; namely, lower serum melatonin levels were present in MS patients compared to healthy controls [22]. Moreover, melatonin has been demonstrated to directly influence *CLOCK* and *ARNTL* expression through post-transcriptional and/or post-translational activities and treatment with melatonin in mice significantly altered gene expression patterns of specific circadian genes [23]. Altered circadian relationship between serum NO, CO2, and UA has also been noticed in MS subjects, suggesting that this alternation may contribute to or reflect the disease processes in multiple sclerosis [24].

Last but not least, clock-related circadian disruption was demonstrated to exist in a mouse model of multiple sclerosis, EAE [25]. Normal fluctuations of *CLOCK* mRNA levels at specific time points during 24h period were significantly reduced in EAE mice, suggesting clock-dependant circadian rhythm disturbances.

Moreover, the link between disturbed circadian rhythms and many different diseases including neurodegenerative diseases has been shown to exist in numerous studies [8–12]. It has been suggested that disturbed circadian rhythms may alter ordered daily cellular molecular metabolic mechanisms and therefore contribute to neurodegeneration on a long term basis [10]. Since cellular metabolic mechanisms are related not specifically to neurodegeneration but to all states of health and disease, circadian rhythms are an important mechanism to be studied in chronic diseases.

The potential limitation of this study is that the association has been investigated in the population of Slave origin; on the other hand, a representative, homogeneous population cohort presents the strength of the study.

In conclusion, our data suggest that variability at *ARNTL* and *CLOCK* gene loci might be associated with MS. Further studies on populations with different genetic background are necessary to validate association.
